# The effect of preoperative fear and anxiety on postoperative pain in surgical patients: a descriptive and correlational study

**DOI:** 10.1007/s11845-026-04412-0

**Published:** 2026-04-27

**Authors:** Derya Gezer, Hamide Şi̇şman

**Affiliations:** 1 Department of Nursing, Faculty of Health Sciences, Tarsus University, Mersin, Türkiye; 2 Kadir Yallagöz School of Health Sciences, Selcuk University, Konya, Türkiye

**Keywords:** Preoperative fear, Preoperative anxiety, Pain, Nursing

## Abstract

**Background:**

Preoperative surgical fear and anxiety, is known to influence postoperative pain perception. The aim of this study is to evaluate the relationship between preoperative surgical fear, surgical anxiety, and postoperative pain severity.

**Methods:**

This descriptive and correlational study included 138 adult patients undergoing elective general surgery at a tertiary care hospital. Preoperative surgical fear and anxiety were assessed one day before surgery using the Surgical Fear Questionnaire (SFQ) and the Surgical Anxiety Questionnaire (SAQ). Postoperative pain intensity was measured using the Visual Analog Scale (VAS) at predefined intervals during the first 24 postoperative hours. Correlation analyses were performed to examine associations between psychological variables and pain intensity.

**Results:**

Patients had mean SFQ and SAQ scores of 34.6±18.1 and 40.4±12.6, respectively. SFQ was weakly but significantly correlated with postoperative pain intensity across all time intervals during the first 24 hours (r = 0.186–0.257, p< 0.05). Similarly, SAQ showed significant positive correlations with postoperative pain at all time points, with slightly stronger associations observed in the early postoperative period (r = 0.232–0.326, p < 0.01). A strong positive correlation was identified between SFQ and SAQ (r = 0.733, p< 0.001). No significant association was found between psychological variables and total analgesic consumption (p > 0.05).

**Conclusion:**

Preoperative surgical fear and anxiety were significantly associated with postoperative pain in general surgery patients, particularly in the early postoperative period. Routine psychological assessment may contribute to improved pain management and perioperative care outcomes.

## Introduction

Surgical interventions directly affect the health outcomes and quality of life of millions of individuals worldwide, with more than 300 million procedures performed annually [1]. For patients, the surgical process represents not only a physical intervention but also an emotionally challenging experience characterized by uncertainty, loss of control, and concerns regarding postoperative recovery [2]. Postoperative pain is a central component of the perioperative period; approximately 80% of surgical patients report acute pain, and fewer than half receive adequate analgesia [3]. Effective postoperative pain management is essential to reduce physiological stress, support early mobilization, prevent complications, and minimize hospital length of stay and healthcare costs [4, 5]. Importantly, inadequately managed acute pain is a major risk factor for chronic postsurgical pain, which affects 10–50% of patients undergoing general surgical procedures [6].

Preoperative anxiety is prevalent among surgical patients, with reported rates ranging from 11% to 80% and higher levels observed in women [7–10]. Elevated anxiety has been associated with increased postoperative pain intensity, higher analgesic requirements, delayed recovery, and an increased likelihood of developing chronic postoperative pain [5, 11–14]. Preoperative anxiety may also increase anesthetic and analgesic drug demands by altering physiological responses, pain perception, and medication metabolism [15]. Moreover, high levels of anxiety have been linked to adverse postoperative outcomes, including delirium and cardiac complications [16].

Surgical fear, a related yet distinct construct, involves concerns about both short-term consequences such as anesthesia, pain, and adverse effects and long-term outcomes including postoperative recovery [17, 18]. Patients may exhibit markedly different levels of fear in response to the same surgical threat, reflecting individual differences in psychological vulnerability and coping capacity [10]. Evidence suggests that preoperative fear, similar to anxiety, may be associated with increased postoperative pain, higher anesthetic and analgesic needs, and impaired physical and psychosocial recovery [17, 19–23]. However, the relationship between preoperative fear and postoperative pain in general surgery patients has not been adequately investigated, and the current understanding within this patient group remains limited [24].

Given that nurses play a pivotal role in preoperative psychological assessment, perioperative education, pain evaluation, and postoperative care, identifying how preoperative fear and anxiety influence postoperative pain is crucial for improving surgical outcomes. In this context, The findings are expected to address a significant gap in the literature and support evidence-based nursing practices to enhance the quality of perioperative care.

## Methods

### Participants and procedure

A descriptive and correlational research design was adopted for this study. Reporting followed the Strengthening the Reporting of Observational Studies in Epidemiology (STROBE) guidelines [25]. The study sample consisted of 138 individuals who underwent elective general surgery in a tertiary-level hospital between October 2024 and June 2025.

**Fig. 1:**
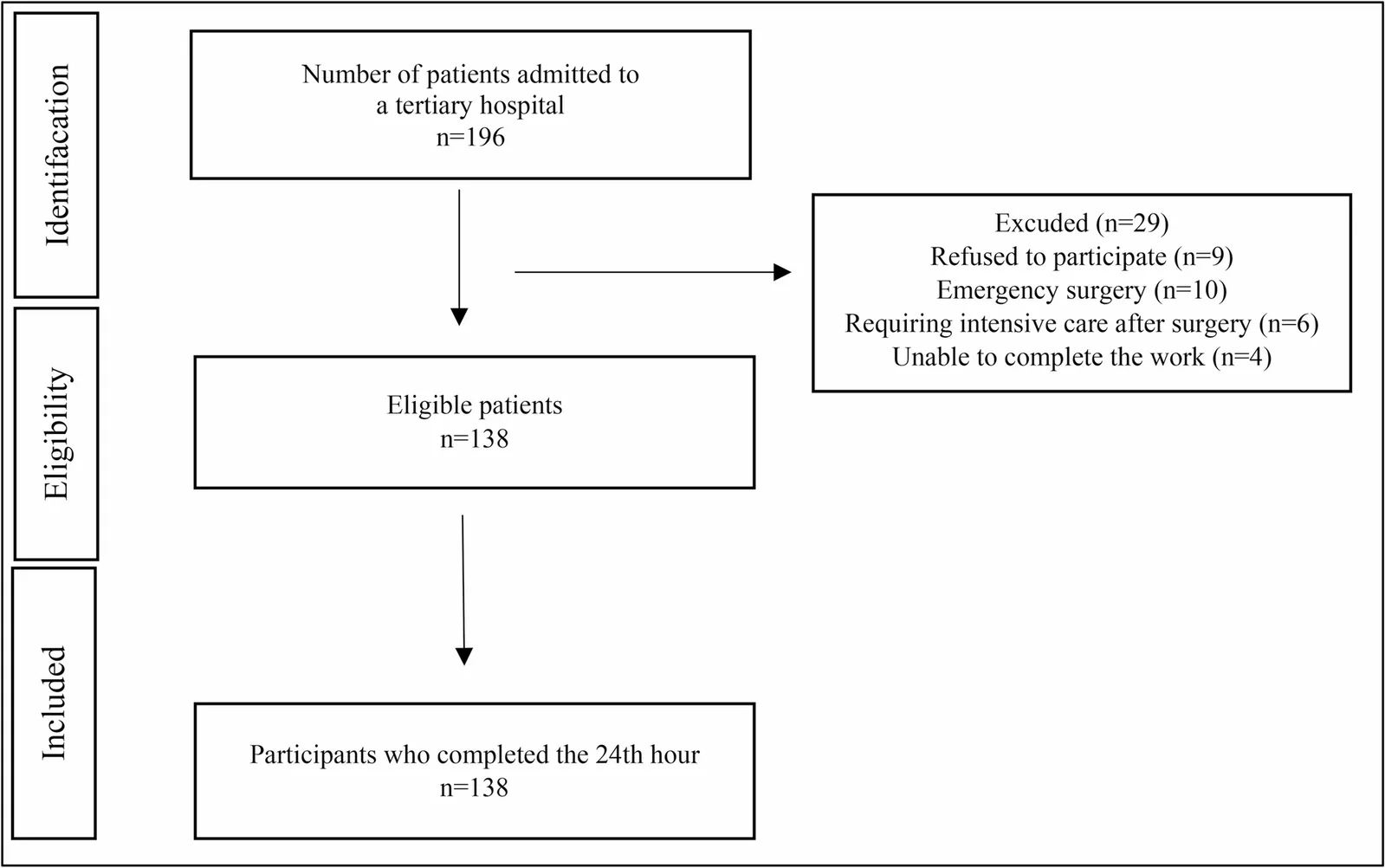
Flow chart of study

According to the Power analysis conducted based on Deniz Doğan’s [19] study (G*Power 3.0.10), it was calculated that an effect size of 0.406, with 95% confidence interval and 95% power, would require 125 patients. Taking into account possible dropouts and confounding variables during the research process, the number was increased by 10% [26]. The sample size for the study was determined to be 138 patients in total. The sampling process is illustrated in Fig. 1.

### Participants

Eligibility criteria for participation included: (a) being 18 years of age or older; (b) scheduled for an elective surgical procedure for the first time; (c) planned to receive general anesthesia; (d) hospitalization on the day preceding surgery; (e) having an anticipated postoperative follow-up period of at least 24 h in the clinic; (f) absence of chronic pain; (g) an American Society of Anesthesiologists (ASA) physical status classification of ≤ 3; and (i) no visual, auditory, or cognitive impairments that could interfere with data collection.

Patients were excluded if they were (a) undergoing emergency surgery or (b) found to have cognitive impairment during the initial screening. Discontinuation criteria included: (a) inability to complete the surgical procedure or the development of severe postoperative complications, (b) the need for an unexpected emergency operation, or (c) postoperative transfer to the intensive care unit.

### Instruments

Study data were collected using a Personal Information Form, Analgesia Monitoring Form, SFQ, SAQ, and VAS, all prepared in accordance with the literature.

The demographic and clinical information form is a 7-item form that examines participants’ socio-demographic and clinical characteristics, including age, gender, marital status, educational level, type of surgery, previous surgical history, and ASA classification [19, 27, 28].

The SFQ consists of 8 items on a numerical scale ranging from 0 to 10. The lowest possible score on the scale is 0, and the highest possible score is 80. High scores on the SFQ indicate high levels of anxiety. The Cronbach’s alpha value for the total scale of the SFQ was reported to be 0.93 [29]. In the present study, the Cronbach’s alpha coefficient of the scale was 0.88.

The SAQ is a 17-item measure using a five-point Likert scale, scored as ‘none-0’, ‘very little-1’, ‘moderate-2’, ‘very much-3’, and ‘extremely-4’. The lowest possible score on the scale is 0, and the highest possible score is 68. As the score increases, the level of surgical anxiety increases. The scale has no cut-off point. The SAQ Cronbach’s alpha coefficient is 0.84 [30]. In this study, the Cronbach’s alpha coefficient was 0.91.

The VAS consists of a 10 cm line where ‘0 = no pain’ and ‘10 = the worst pain imaginable’. An increase in the score indicates an increase in pain intensity [31]. The VAS is recognised as a valid and reliable tool for clinical pain assessment.

The pain analgesic monitoring form documents the severity of pain experienced by patients after surgery and the type of analgesic administered [5, 12, 19].

## Data collection procedure

Data was collected from participants who met the eligibility criteria through face-to-face interviews and structured observations conducted by the researcher. Data collection began one day before surgery and continued for the first 24 h after surgery. On the morning of surgery, demographic and clinical information was obtained from patients in their hospital rooms. Subsequently, the SFQ and SAQ were administered. At this stage, patients were also informed that their post-operative pain levels would be assessed. Pain measurements were performed immediately after the patient returned to the clinic postoperatively and for 24 h postoperatively, as part of routine clinical care. Pain intensity and the type and amount of analgesics prescribed by the doctor were obtained from the patient follow-up chart records. In the data evaluation process of the study, the average pain levels of the patients were given based on the 0–2, 2–4, 4–8, 8–12, and 12–24 h intervals. To minimise potential bias, the person responsible for data collection and the person conducting the statistical analyses were independent of each other.

### Ethical considerations

Approval for the study was granted by the Non-Interventional Clinical Research Ethics Committee (Date/No: 06.09.2024-147 /80) and the administration of the university hospital (Date/No: 24.10.2024–1136864). All procedures were carried out in accordance with the ethical principles outlined in the Declaration of Helsinki. Prior to data collection, participants were informed about the aim of the study, assured that their participation was voluntary and anonymous, and reminded of their right to withdraw at any stage without any consequences.

### Data analysis

The data obtained in the study were analysed using the SPSS (Statistical Package for Social Sciences) for Windows 25.0 programme. Descriptive statistical methods (frequency, percentage, mean, standard deviation) were used when evaluating the data. The normality of the data was evaluated using the Kolmogorov–Smirnov test, and since the data did not meet normal distribution assumptions, nonparametric tests (Kruskal–Wallis and Mann–Whitney U) were employed. Pearson Correlation analysis was applied to find the relationship between the dependent and independent variables. A correlation coefficient between 0.80 and 1.0 indicates a very strong correlation; between 0.60 and 0.79 indicates a strong correlation; between 0.40 and 0.59 indicates a moderate correlation; between 0.20 and 0.39 indicates a weak correlation; and between 0 and 0.19 as a very weak correlation [32]. The results were evaluated at a 95% confidence interval and a significance level of *p* < 0.05.

## Results

Table 1 presents the demographic data of the participants. The mean age of the participants was 50 ± 15 (Min = 18, Max = 85), 52.2% were female, 89.1% were married, and 34.1% were primary school graduates. When asked about the presence of chronic disease, 39.9% had a chronic disease, and gastrointestinal surgery was the most common procedure performed (26.8%). 46.4% of participants had surgical experience, and 77.5% had an ASA score of 2 (Table 1).

**Table 1:** Sociodemographic and clinical variables of participants (*n* = 138)

Variables	Min-Max	
Age ($\:\stackrel{-}{\boldsymbol{X}}$±SD, 50 ± 15)(year)	18–85	
Gender	**n**	**%**
Male	66	47.8
Female	72	52.2
Marital status		
Married	123	89.1
Single	15	10.9
Chronic disease		
Yes	55	39.9
No	83	60.1
Education		
İlliterate	12	8.7
Literate	25	18.1
Primary school	47	34.1
Secondary school	28	20.3
University	26	18.8
Surgery Type		
Cholelithiasis	29	21
Breast and endocrine surgery	35	25.4
GIS surgery*	37	26.8
Hernia Surgery (4)	30	21.7
Another (perianal fissure, ileus, anal incontinence)	7	5.1
Surgery experience		
Yes	64	46.4
No	74	53.6
ASA Score		
1	28	20.3
2	107	77.5
3	3	2.2

Data are expressed as numbers (n) and frequency (%)

* Gastrointestinal system surgery

Table 2 examines the participants’ pain levels and analgesic usage characteristics. The highest postoperative pain was in the 0–2 h interval (4.73 ± 2.35), and the lowest was in the 12–24 h interval (0.91 ± 1.21). The highest number of analgesic drug applications to participants was administered between 0 and 2 h (92%), and the lowest between 4 and 8 h (46.4%). When the type of analgesic administered was evaluated, it was found that the most commonly used analgesic type between 0 and 2 h was Paracetamol (IV) (66.7%). However, there is no significant relationship between SFQ and SAQ levels and total analgesic use (*p* > 0,05)(Table 2).

**Table 2:** Participants’ pain level and analgesic use characteristics

Variables	0-2nd hours			2-4th hours		4-8th hours			8-12th hours			12-24th hours	
	$\:\stackrel{-}{\boldsymbol{X}}$±SD			$\:\stackrel{-}{\boldsymbol{X}}$±SD		$\:\stackrel{-}{\boldsymbol{X}}$±SD			$\:\stackrel{-}{\boldsymbol{X}}$±SD			$\:\stackrel{-}{\boldsymbol{X}}$±SD	
Pain intensity	4.73 ± 2.35			3 ± 2.04		2.4 ± 1.92			2.13 ± 1.77			0.91 ± 1.21	
Analgesic type	***n***		***%***	***n***	***%***	***n***	***%***		***n***	***%***		***n***	***%***
Not implemented	11		8	92	66.7	74	53.6		84	61		23	16.7
Paracetamol (IV)	92		66.6	19	13.8	26	18.8		30	21.7		73	52.9
Diclofenac sodium (IM)	20		14.5	10	7.2	8	5.8		10	7.2		33	23.9
Ibuprofen (IV)	4		2.9	14	10.1	30	21.8		13	9.4		4	2.9
Tramadol hidroklorür (IV)	11		8	3	2.2	0	0		1	0.7		5	3.6
								**SFQ**			**SAQ**		
Total Analgesic number		*Pearson*						0.150			0.124		
		*p*						0.26			0.35		

*IV* Intravenöz, *IM* Intramuscular

Data are expressed as numbers (n) and frequency (%)

Pearson Correlation Analysis

The participants’ average SFQ score was 34.6 ± 18.1, while their average SAQ score was 40.4 ± 12.6. A statistically significant relationship was found between educational status and mean SFQ scores, with the highest mean SFQ score found in the literate group (44.5 ± 11.5) and the lowest mean score found in participants who had completed secondary education (30.8 ± 17.8) (F = 2.59, *p* = 0.04). A statistically significant relationship was found between diagnosis and mean SFQ scores, with the highest mean SFQ score found in the gastric cancer group (44.1 ± 11.8) and the lowest mean score found in participants diagnosed with hernia (26.1 ± 22.4) (F = 3.03, *p* = 0.00). A statistically significant relationship was found between surgery experience and mean SFQ scores, with the highest mean SFQ score found in the group without surgery experience (38.2 ± 15.9) (t=-2.59, *p* = 0.01). A statistically significant relationship was found between educational status and SAQ mean scores, with the highest SAQ mean score found in the literate group (48.7 ± 6.94) and the lowest mean score found in participants who had completed secondary school (36.5 ± 12.1) (F = 3.94, *p* = 0.00) (Table 3).

**Table 3:** Comparison of surgical fear and surgical anxiety scores by patient characteristics

Variables	*n*	SFQ $\:\stackrel{-}{\boldsymbol{X}}$±SD	Test	Significant Differences	SAQ $\:\stackrel{-}{\boldsymbol{X}}$±SD	Test	Significant Differences
Total		34.6 ± 18.1			40.4 ± 12.6		
Sex							
Male (1)	66	32.9 ± 19.9	Z=-0.224^®^ *p* = 0.82	-	38.5 ± 12.6	Z=-1.320 *p* = 0.18	
Female (2)	72	36.1 ± 16.5			42.3 ± 12.3		
Marital status							
Married (1)	123	34.8 ± 18.1	Z=-0.308^®^ *p* = 0.75	-	40.1 ± 12.3	Z=-1.468 *p* = 0.142	-
Single (2)	15	33.1 ± 19.7			44.3 ± 14.8		
Education							
İlliterate (1)	12	35.9 ± 18.1	ꭓ2 = 12.491^©^ *p* = 0.01^**a**^	1–2 2–3,4,5	40.9 ± 13.9	ꭓ2 = 15.295 *p* = 0.00^**a**^	1–2 2–3,4,5
Literate (2)	25	44.5 ± 11.5			48.7 ± 6.94		
Primary School (3)	47	32.9 ± 19.3			38.6 ± 13.1		
High School (4)	28	30.8 ± 17.8			36.5 ± 12.1		
University (5)	26	31.2 ± 19.5			39.9 ± 13.2		
Chronic disease							
Yes (1)	55	37.4 ± 16.5	Z=-1.146^®^ *p* = 0.25	-	41.5 ± 12.3	Z=-0.681 *p* = 0.49	-
No (2)	83	32.6 ± 19.1			39.7 ± 12.7		
Surgery Type							
Cholelithiasis (1)	29	32.4 ± 18.2	ꭓ2 = 17.165^©^ *p* = 0.00^**a**^	3 − 1,2,4,5	38.5 ± 12.6	ꭓ2 = 9.358 *p* = 0.05	-
Breast and endocrine surgery (2)	35	31.2 ± 16.5			40 ± 12.1		
GIS surgery (3)	37	44 ± 10.5			45.2 ± 9.7		
Hernia Surgery (4)	30	29 ± 21.4			38 ± 14.5		
Another (perianal fissure, ileus, anal incontinence) (5)	7	36 ± 27.4			36.1 ± 15.1		
Surgery experience							
Yes (1)	64	30.3 ± 19.8	Z=-3.092^®^ *p* = 0.00^**a**^	2 > 1	38.8 ± 13.5	Z=-1.815 *p* = 0.06	-
No (2)	74	38.2 ± 15.9			41.9 ± 11.6		
ASA Score							
1 (1)	28	39.2 ± 15.5	ꭓ2 = 3.881^©^ *p* = 0.14	-	42.1 ± 1.99	ꭓ2 = 2.368 *p* = 0.30	-
2 (2)	107	33 ± 18.4			40.3 ± 13.1		
3 (3)	3	38 ± 10			31.3 ± 0.59		

^®^Mann-Whitney U test (Z-table value)

^©^Kruskall-Wallis H” test (χ2-table value)

^a^Statistically significant differences were observed between illiterate and literate participants, and between the literate group and those with higher educational attainment

There is no statistically significant relationship between age and SFQ and SAQ. There is a positive correlation between the SFQ mean score and the mean pain scores in the 0–2, 2–4, 4–8, 8–12, and 12–24 There is a weak positive correlation between the SFQ mean score and the mean pain scores in the 0–2, 2–4, 4–8, 8–12, and 12–24 h intervals (*r* = 0.257, *r* = 0.235, *r* = 0.217, *r* = 0.250, *r* = 0.186, respectively). In other words, as participants’ SFQ scores increase, their pain levels also increase. There was a positive correlation between the SAQ score average and the pain levels in the 0–2, 2–4, 4–8, 8–12, and 12–24 h intervals after surgery (*r* = 0.232, *r* = 0.299, *r* = 0.315, *r* = 0.237, *r* = 0.326, respectively). In other words, as participants’ SFQ and SAQ scores increased, their pain levels also increased. Furthermore, there is a high level of positive and statistically significant correlation between the SFQ and SAQ (*r* = 0.733, *p* < 0.001) (Table 4).

**Table 4:** Associations of surgical fear and anxiety with pain intensity during the first 24 h postoperatively

Pain Intensity		Surgical Fear	Surgical Anxiety
Age	*Pearson*	0.101	0.049
	*p*	0.241	0.567
0-2nd hours	*Pearson*	0.257	0.326
	*p*	**0**,00*	0.00*
2-4th hours	*Pearson*	0.235	0.299
	*p*	0.00*	0.00*
4-8th hours	*Pearson*	0.217	0.315
	*p*	0.01*	0.00*
8-12th hours	*Pearson*	0.250	0.237
	*p*	0.00*	0.00*
12-24th hours	*Pearson*	0.186	0.232
	*p*	0.02*	0.00*
Surgical Fear	*Pearson*	0.733	**1**
	*p*	0.00*	**-**

*Pearson Correlation Analysis

P-values in bold are statistically significant (*p* < 0.05).* *p* < 0.01

## Discussion

This study examined the relationship between preoperative surgical fear and surgical anxiety levels in general surgery patients and postoperative pain. According to our findings, participants’ surgical fear and surgical anxiety levels were moderate to high. This result indicates that the surgical process places not only a physical but also a significant psychological burden on patients.

The literature reports that 50% to 90% of patients scheduled for surgery experience preoperative fear [8, 33, 34], while preoperative anxiety has a prevalence rate of approximately 48%. The moderate SFQ mean score obtained in our study [34.6 ± 18.1] is consistent with the values reported in the literature [19, 34–37]. Similarly, the SAQ mean scores [40.4 ± 12.6] being above moderate indicate that anxiety in surgical patients is a clinically significant problem. Similar levels of surgical anxiety are also found in the literature [5, 10, 38]. The fundamental dimensions of fear contributing to preoperative anxiety include fear of the unknown, feeling ill, anesthesia complications, postoperative pain, and fear of death [39, 40]. Considering these factors, it is expected that the surgical process will increase fear and anxiety.

Following these general findings, when individual factors affecting surgical fear and anxiety were evaluated, educational level, type of surgery, and previous surgical experience were among the risk factors frequently reported in the literature [27, 41–43]. Our study found that fear and anxiety levels differed according to educational level; fear and anxiety levels decreased as educational level increased, with the lowest levels found among high school graduates. The association of educational level with surgical fear and anxiety is consistent with the literature [38, 44, 45]. However, in addition to studies reporting that anxiety levels increase with increasing educational level [46, 47], there are also studies suggesting that educational level is not a reliable indicator of preoperative anxiety [34, 36]. This heterogeneity suggests that educational level alone is not a determinant of anxiety and that individual, cultural, and clinical characteristics may play an important role in this relationship.

In this study, it was determined that preoperative surgical anxiety levels differed according to the type of planned surgical procedure, with the highest anxiety levels observed in patients scheduled for gastrointestinal system (GIS) surgery. The literature reports a significant increase in anxiety levels prior to major surgery [41–43]. In this regard, the fact that GI surgeries generally involve larger anatomical areas, the higher perceived risk of complications, and the greater uncertainty regarding the postoperative recovery process may explain the higher levels of surgical anxiety observed in this patient group.

However, it has been found that previous surgical experience significantly reduces preoperative anxiety in patients. This finding is consistent with studies reporting that having previously undergone anaesthesia or surgical intervention reduces the development of preoperative anxiety [34, 41, 46]. This can be explained by patients having gained experience with the operating theatre environment, anaesthesia procedures, and the postoperative pain process.

It is known that psychological stress increases pain perception in the acute postoperative period; this effect occurs through both physiological responses and cognitive processes [48]. The effect of psychological factors on pain mechanisms is multidimensional, with fear and anxiety being among the strongest individual predictors of postoperative pain [17, 45, 49, 50]. The present study found that surgical fear and anxiety showed consistent and positive relationships with pain during all time periods in the first 24 h postoperatively. Similarly, recent meta-analyses report that preoperative anxiety is significantly related to postoperative pain levels [6, 12, 15, 51]. These findings are consistent with the literature on surgical anxiety [5, 10, 14, 16, 52] and surgical fear [17, 19, 45, 53], which demonstrates that the preoperative psychological state modulates the physiological pain response.

The highest correlations observed in our study during the early postoperative period (0–2 and 4–8 h) support the decisive role of psychological factors in acute pain perception. These relationships can be explained by biopsychosocial mechanisms such as increased sympathetic activation due to the stress response, hypervigilance, cognitive focus on pain, impaired physiological regulation, and decreased pain thresholds [22]. Therefore, it can be said that surgical fear and anxiety increase postoperative pain both directly, by enhancing pain perception, and indirectly, by intensifying the stress response.

However, it is noteworthy that the study did not find a significant relationship between surgical fear and anxiety levels and total analgesic use. This finding contradicts studies in the literature reporting that increasing surgical fear and anxiety are associated with increased analgesic use [19, 35]. This result, which at first glance appears to contradict the literature suggesting that psychological factors can influence postoperative pain perception, can be explained by the fact that analgesic use is mostly planned according to standard clinical protocols, the type of surgical procedure, anaesthesia, and pain management approaches. In other words, although patients’ subjective psychological states may influence their pain experience, this may not have been directly reflected in analgesic dosage or frequency of use. In addition, total analgesic consumption should be interpreted cautiously, as different postoperative analgesics vary in mechanism of action, potency, and clinical indication. Therefore, recording analgesic use only as a total amount may have masked potential differences in analgesic requirements related to preoperative fear and anxiety.

In the present study, a high level of positive linear correlation was found between the surgical fear experienced by patients and surgical anxiety. This result is consistent with the literature [19, 33, 42]. Perceived risk associated with surgery, feelings of loss of control during surgery, and negative expectations regarding surgical outcomes may contribute to an increase in both fear and anxiety [51]. Expectations of potential complications or death intensify anxiety, increasing stress related to the surgical process [14]. These findings indicate that surgical fear and anxiety are mutually reinforcing emotional states closely related to the surgical process.

When all these results are considered together, preoperative fear and anxiety not only increase postoperative pain; but also trigger physiological (increased sympathetic activation, cortisol response), behavioural (excessive focus on pain), and neuropsychological (impaired pain modulation) mechanisms that can negatively affect the recovery process. This study contributes to the literature by revealing the time-dependent effect of surgical fear and anxiety on pain in the early postoperative period in general surgery patients. Our findings show that assessing preoperative surgical fear and anxiety in surgical patients is critical for predicting and managing postoperative pain. Identifying levels of surgical fear and anxiety early on suggests that targeted interventions such as psychoeducation, relaxation techniques, cognitive-behavioural interventions, and preoperative counselling for high-risk patients may improve pain management. In nursing practice, routine preoperative screening for surgical fear and anxiety may help identify patients who require closer monitoring and individualized supportive care. Based on these assessments, nurses can implement practical interventions such as structured preoperative education, therapeutic communication, emotional support, and relaxation guidance to improve perioperative outcomes. These findings contribute to clinical practice in terms of both individualising nursing care and more effectively implementing multimodal analgesia protocols.

### Study limitations

This study has several limitations. First, it was conducted in a single center and included only general surgery patients, which may limit the generalizability of the findings to other surgical specialties, clinical settings, and broader patient populations. Therefore, future multicenter studies involving more diverse patient groups are recommended to strengthen the external validity of the findings. Another limitation of this study is that postoperative analgesic use was recorded only as total consumption, without detailed documentation of specific analgesic agents and dosages (mg). Because different analgesics vary in mechanism of action, potency, and clinical effect, this may have limited precise quantitative comparison and may have obscured the relationship between psychological factors, such as surgical fear and anxiety, and analgesic consumption. Furthermore, the use of standard analgesic protocols may have reduced the extent to which individual pain perception was reflected in analgesic use.

Furthermore, the potential influence of non-pharmacological nursing interventions (e.g., positioning, relaxation, and information provision) on analgesic requirements was not evaluated. Future multicenter and longitudinal studies involving diverse surgical populations are needed to clarify the causal pathways between preoperative psychological factors and both acute and persistent postoperative pain. Incorporating standardized analgesic consumption measures, physiological stress markers, and recovery outcomes, as well as randomized controlled trials of preoperative psychological interventions, may strengthen the evidence base for postoperative pain management.

## Conclusion

This study has demonstrated that preoperative surgical fear and anxiety in general surgery patients have a significant effect on postoperative pain. It was found that patients with high levels of fear and anxiety in the preoperative period experienced more severe pain in the postoperative period. These findings indicate that the preoperative psychological state is an important clinical variable affecting postoperative pain outcomes. The results obtained emphasise the critical role of surgical nursing in the perioperative care process. Surgical nurses play a central role in the early identification and management of fear and anxiety in the preoperative period. In this context, making psychological assessment a routine part of care may positively affect postoperative pain control and the recovery process. Consequently, addressing preoperative psychological stress as a fundamental component of multidisciplinary surgical care and ensuring that surgical nurses take on an active and therapeutic role in this process are considered important requirements in clinical practice.
